# Imprint cytology on microcalcifications excised by Vacuum-Assisted Breast Biopsy: A rapid preliminary diagnosis

**DOI:** 10.1186/1477-7819-5-40

**Published:** 2007-04-03

**Authors:** Maria Fotou, Vassiliki Oikonomou, Flora Zagouri, Theodoros N Sergentanis, Afroditi Nonni, Pauline Athanassiadou, Theodora Drouveli, Efstratios atsouris, Evagelia Kotzia, George C Zografos

**Affiliations:** 1 Department of Pathology, School of Medicine, Athens University, Greece; 2 Department of Pathology, Laboratory Unit, School of Medicine, Athens University, Greece; 3 Department of Cytology, Hippocratio Hospital, Athens, Greece; 4 Department of Surgery, Breast Unit, 1st Department of Surgery, School of Medicine, Athens University, Greece

## Abstract

**Background:**

To evaluate imprint cytology in the context of specimens with microcalcifications derived from Vacuum-Assisted Breast Biopsy (VABB).

**Patients and methods:**

A total of 93 women with microcalcifications BI-RADS 3 and 4 underwent VABB and imprint samples were examined. VABB was performed on Fischer's table using 11-gauge Mammotome vacuum probes. A mammogram of the cores after the procedure confirmed the excision of microcalcifications. For the application of imprint cytology, the cores with microcalcifications confirmed by mammogram were gently rolled against glass microscope slides and thus imprint smears were made. For rapid preliminary diagnosis Diff-Quick stain, modified Papanicolaou stain and May Grunwald Giemsa were used. Afterwards, the core was dipped into a CytoRich Red Collection fluid for a few seconds in order to obtain samples with the use of the specimen wash. After the completion of cytological procedures, the core was prepared for routine histological study. The pathologist was blind to the preliminary cytological results. The cytological and pathological diagnoses were comparatively evaluated.

**Results:**

According to the pathological examination, 73 lesions were benign, 15 lesions were carcinomas (12 ductal carcinomas *in situ*, 3 invasive ductal carcinomas), and 5 lesions were precursor: 3 cases of atypical ductal hyperplasia (ADH) and 2 cases of lobular neoplasia (LN). The observed sensitivity and specificity of the cytological imprints for cancer were 100% (one-sided, 97.5% CI: 78.2%–100%). Only one case of ADH could be detected by imprint cytology. Neither of the two LN cases was detected by the imprints. The imprints were uninformative in 11 out of 93 cases (11.8%). There was no uninformative case among women with malignancy.

**Conclusion:**

Imprint cytology provides a rapid, accurate preliminary diagnosis in a few minutes. This method might contribute to the diagnosis of early breast cancer and possibly attenuates patients' anxiety.

## Background

The increased use of mammograms has led to the frequent detection of breast lesions, which in turn require further evaluation [1,2]. The most common mammographic abnormalities found on screening examinations are microcalcifications [3,4]. Radiographic mammary microcalcifications occur in 30–50% of breast cancer cases and constitute one of the most important diagnostic markers in both benign and malignant lesions of the breast [3,4]. When suspicious microcalcifications appear on a mammogram, a needle localization core biopsy is recommended, so that breast tissue can be removed and examined under a microscope by a pathologist. Although microcalcifications are the commonest radiological feature of ductal carcinoma *in situ *(DCIS), about 90% of women with abnormal microcalcifications do not have breast cancer; this highlights the importance of a safe and efficient evaluation [5,6].

According to the Breast Imaging Reporting and Data System (BI-RADS) of the American College of Radiology, a biopsy is suggested for lesions classified as category 4 (suspicious), and category 5 (highly suggestive of malignancy) [7-9]. Additionally, a biopsy can be performed in category 3 lesions if the patient or the referring physician insists [9,10]. Owing to the broad spectrum of the BI-RADS 4 category and the corresponding wide range of malignancy incidence within it, this category is subdivided into group 4A, 4B and 4C [9-11].

To establish a preoperative diagnosis, excisional biopsies, core needle biopsies [12-14] and vacuum-assisted breast biopsies (VABB) have been used [15,16]. VABB on the Fischer's table has become an integral part of the work-up of patients with suspicious breast lesions, as it is established as a safe and effective technique providing a minimally invasive, faster, and less painful method for sampling microcalcifications seen on mammograms [17-23].

Recently, core imprint cytology has been shown to have a high sensitivity in diagnosing malignancy. Imprint cytology is a technique that is used in our centre for breast specimens obtained after the performance of VABB. As far as breast cancer is concerned, imprint cytology has been extensively discussed at the intraoperative level, for the assessment of sentinel lymph nodes [24-27], and secondarily at the preoperative level, i.e. diagnosis of breast carcinoma. The significance, rapidity and reliability of the method have been documented in the context of needle core biopsy [28-30]. However, to our knowledge, no studies have focused on imprint cytology specimens derived from VABB. The purpose of this study is to evaluate the application on imprint cytology on cores with microcalcifications derived from VABB.

## Patients and methods

We describe our experience based on 93 procedures performed for microcalcifications from January 2005 to September 2006, in our Breast Unit on women aged 35–75 years (50.66 ± 9.10, mean ± SD).

Before the VABB, all patients were evaluated by one of the two radiologists assigned to the Breast Imaging Section. For lesions categorized as BI-RADS 3, follow-up was generally recommended. However, VABB was performed in cases where family history was strongly positive or when the patient and referring physician expressed particular concern. In our unit, BI-RADS category 5 cases were directly submitted to open surgical biopsy (operation) in view of the great likelihood of cancer. VABB was performed on digital prone table (Mammotest, Fischer Imaging, Denver, CO, USA) using 11-gauge Mammotome vacuum probes, under local anesthesia. The examination proceeded according to a standard protocol to assure quality control. The surgeon attempted to procure 24 or more cores in each instance. A mammogram of the cores after the procedure confirmed the excision of microcalcifications.

For the application of imprint cytology only the cores with microcalcifications confirmed by mammogram after the procedure were used. On the other hand, for the establishment of the final pathological diagnosis, all the cores obtained by VABB were examined.

The cores were gently rolled against glass microscope slides and thus imprint smears were made. For rapid preliminary diagnosis: i) air dried smears were stained with a Diff-Quick stain, ii) smears were fixed in 95% ethyl alcohol for 20 min and stained with a modified Papanicolaou stain and iii) air dried smears were stained by May Grunwald Giemsa. Afterwards, the cores were dipped into a CytoRich Red Collection fluid for a few seconds in order to obtain samples with the use of the specimen wash. After the completion of cytological procedures, the cores were plunged into formalin and were prepared for routine pathological study. The pathologist was blind to the preliminary cytological results.

Permission has been obtained from the local institutional review board for publication of the findings summarized in this study.

## Results

The sample comprised 93 specimens. Among them, 31.2% (29 out of 93) came from BI-RADS 3 lesions, 45.2% (42 out of 93) from BI-RADS 4A lesions, 22.5% (21 out of 93) from BI-RADS 4B lesions and 1.1% from BI-RADS 4C lesions (1 case). According to the pathological examination, 73 lesions were benign (Table 1) and 15 lesions were malignant; the latter comprised 12 DCIS cases and 3 IDC cases. Five lesions were precursor (3 cases of ADH and 2 cases of LN).

**Table 1:** Spectrum of benign lesions in the sample

	**BIRADS classification**			
**Histopathological diagnosis**	**BI-RADS 3**	**BI-RADS 4A**	**BI-RADS 4B**	***Total***

**Fibrocystic change**^1^	10	8	6	24
**Fibroadenoma**	2	3	1	6
**Sclerosing adenosis**	8	7	0	15
**Adenosis**	4	3	4	11
**Papilloma**	0	3	2	5
**Ductal ectasia**	2	3	2	7
**Epitheliosis with atypia**	1	1	0	2
**Monckeberg calcific sclerosis**	0	0	1	1
**Periductal mastitis**	1	1	0	2

***Total***	28	29	16	73

^1^As the sole finding; when fibrocystic changes coexisted with other, more important disorders, the case was attributed to the latter.

**Figure 1:**
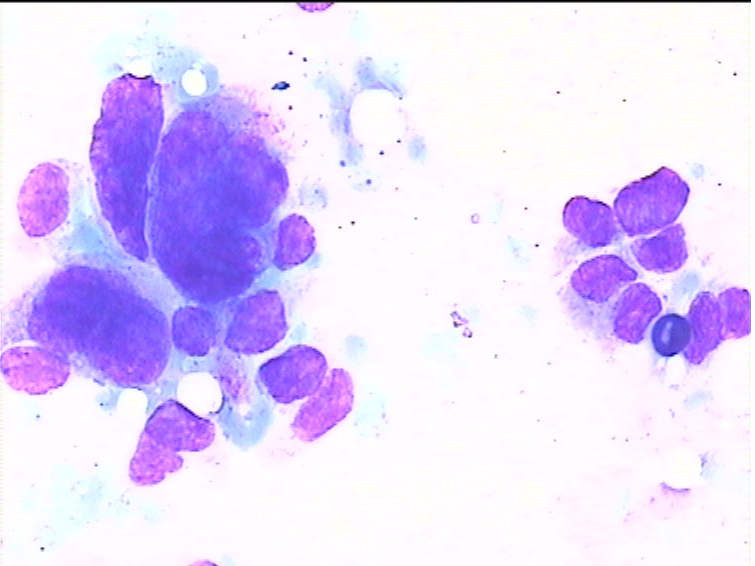
Cytology imprint derived from a malignant lesion.

**Figure 2:**
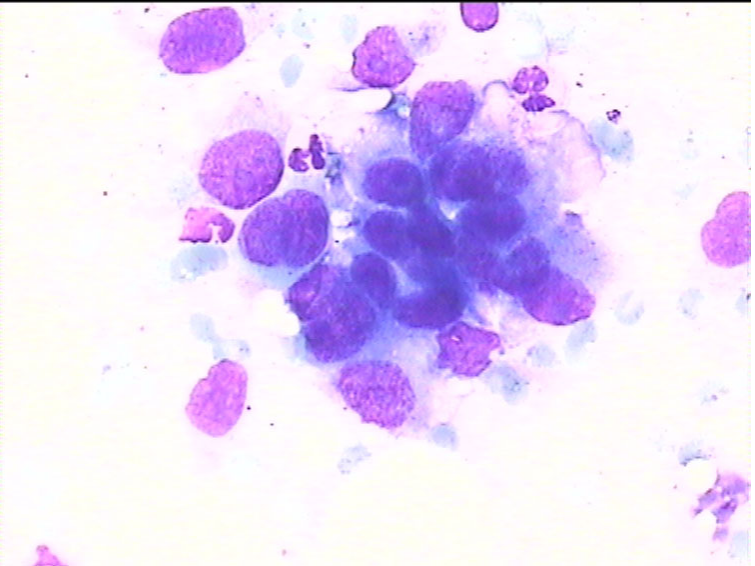
Cytology imprint derived from another malignant lesion.

**Figure 3:**
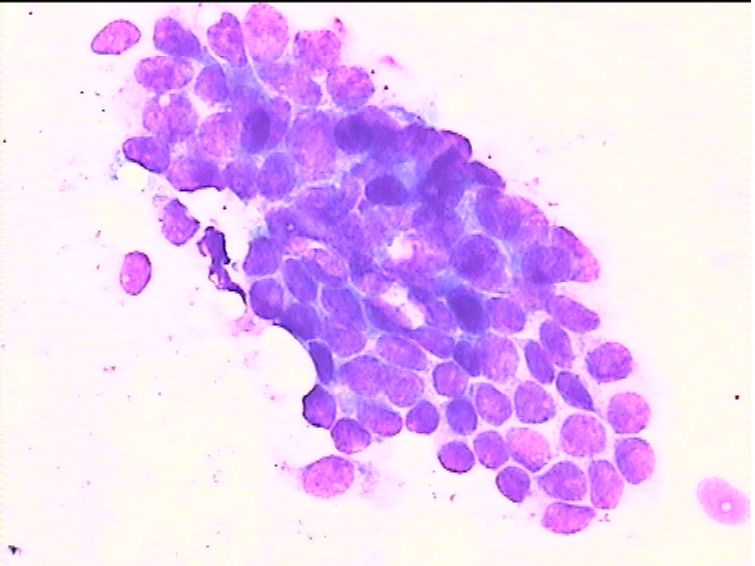
Cytology imprint derived from a benign lesion.

The results of imprint cytology vs. the pathological results are shown in Table 2. Representative cases are shown in Figures 1, 2, 3. The observed sensitivity of the cytological imprints for cancer was 100% (15 true positive/15 malignant cases), since no false negative results appeared. Similarly, the specificity of the method for cancer was 100%, given that there were no false positive cytological diagnoses. Due to the fact that there was total identity of results between the two methods, only the one-sided, 97.5% confidence interval could be calculated for both proportions (sensitivity and specificity), and it was 78.2%–100%.

**Table 2:** MicrocalcificationCytological and pathological results along with BI-RADS classification

	**Cytological results (with negative pathological results)**			**Cytological results (with positive pathological results)**			***Total***
		
	Benign (true-negative)	Malignant (false-positive)	Uninformative	Benign (false-negative)	True-positive	Uninformative	
**BI-RADS 3**							
Benign	24	0	4	-	-	-	28
Cancer	-	-	-	0	1	0	1
							
**BI-RADS 4A**							
Benign	27	0	2	-	-	-	29
Precursor	-	-	-	3	1^a^	1 ^b^	5
Cancer	-	-	-	0	8	0	8
							
**BI-RADS 4B**							
Benign	11	0	5	-	-	-	16
Cancer	-	-	-	0	5	0	5
							
**BI-RADS 4C**							
Cancer	-	-	-	0	1	0	1

Total	62	0	11	3	16	1	93

^a ^this case is an ADH lesion

^b ^this case is a LN lesion

As far as precursor lesions are concerned, only one case of ADH could be detected by imprint cytology, i.e. 33.3% of ADH cases [95%CI: 0.8%–90.6%]; the other two cases yielded false-negative imprints (i.e. having a benign appearance). Neither of the 2 LN cases was detected by the imprints, the first yielding an uninformative result and the other one a false negative (having a benign appearance) cytological diagnosis.

Finally, the cytological imprints were uninformative in 11 out of 93 cases (11.8%), irrespectively of BI-RADS classification or histopathological diagnosis. 13.8% (4 out of 29) of BI-RADS 3 cases yielded uninformative specimens, 4.8% (2 out of 42) of BI-RADS 4A cases were uninformative, and the respective percentage was 23.8% (5 out of 21) for BI-RADS 4B lesions. It should be stressed that no uninformative cases existed within the malignant subgroup.

## Discussion

The importance of anxiety in the management of breast patients has been well demonstrated in the literature, even during mild medical procedures [32,33]. It has been reported that patients experience clinically marked levels of anxiety while they undergo breast biopsy, irrespectively of whether it is an open biopsy or core needle biopsy [34-36]. Interestingly, the use of oral anxiolytic medication has been proposed, in order to reduce anxiety women experience [37].

An effective means of reducing anxiety could be a reliable immediate diagnosis after VABB. A rapid answer could represent a valuable relief for women, given that a pathological diagnosis might take four to seven days to be made. Imprint cytology is the technique that could give a preliminary diagnosis within a few minutes.

The sensitivity and the specificity of the method regarding malignancies seem to be absolutely satisfactory, since both were 100% in the sample. The lack of false positive diagnoses (specificity 100%) implicates that imprint use is not capable of imposing unjustified additional anxiety upon the patients. Additionally, the lack of false negative results indicates that no tumors escape the method. It is worth mentioning that uninformative samples were not present among malignant cases and thus did not interfere with cancer detection. Thus, a malignant diagnosis by imprint cytology could be considered almost definitive and the patients should be psychologically prepared for the subsequent operation.

According to our sample, the main drawback of the method pertains to the detection of precursor breast lesions (ADH, LN). Due to the small number of ADH cases, the absolute percentage of lesions detected by imprint cytology (33.3%) should be interpreted with caution. This is reflected upon the extremely large confidence interval, which leaves open all possibilities for the future development of the method. The clinicians should bear in mind the problems of the method with respect to precursor lesions, and should thus not over-reassure the patient, unless the final pathologic diagnosis is established. Further studies encompassing larger samples and focusing on precursor lesions are needed.

## Conclusion

This study is the first one to describe the application of imprint cytology in the context of microcalcifications excised by VABB. It demonstrates the very satisfactory sensitivity and specificity of the method. Its capability to provide a rapid preliminary diagnosis in a few seconds and a final cytological diagnosis the day after renders it particularly attractive. Further studies, on larger samples are needed to establish the method in the common clinical practice.

## List of abbreviations used

VABB = Vacuum-assisted breast biopsy

BI- RADS = Breast Imaging Reporting and Data System

DCIS = ductal carcinoma in situ

IDC = invasive ductal carcinoma

ADH = atypical ductal hyperplasia

LN = lobular neoplasia

## Competing interests

The author(s) declare that they have no competing interest.

## Authors' contributions

**MF***: *conception of the idea of the study, preparation and evaluation of imprint cytological smears

**VO***: *conception of the idea of the study, preparation and evaluation of imprint cytological smears

**FZ***: *writing of the manuscript, performing VABB, comparative evaluation of pathological and cytological results

**TNS***: *statistical analysis, writing of the manuscript, contributing to the design of the study

**AN***: *establishment of pathological diagnosis

**PA***: *revising the manuscript for important intellectual content

**TD***: *contribution to the preparation of cytological smears, evaluation of the analyzed findings

**EP***: *revising the manuscript for important intellectual content, gave the final approval of the version to be published

**EK***: *revising the manuscript for important intellectual content

**GCZ***: *design of the study, responsible for the performance of VABB in all patients
